# Supplementary figures and images for: Iron chelation improves ineffective erythropoiesis and iron overload in myelodysplastic syndrome mice
Source: eLife. 2023 Dec 28;12:e83103. doi: 10.7554/eLife.83103 (PMC10754500; doi:10.7554/eLife.83103)

## Slide 1
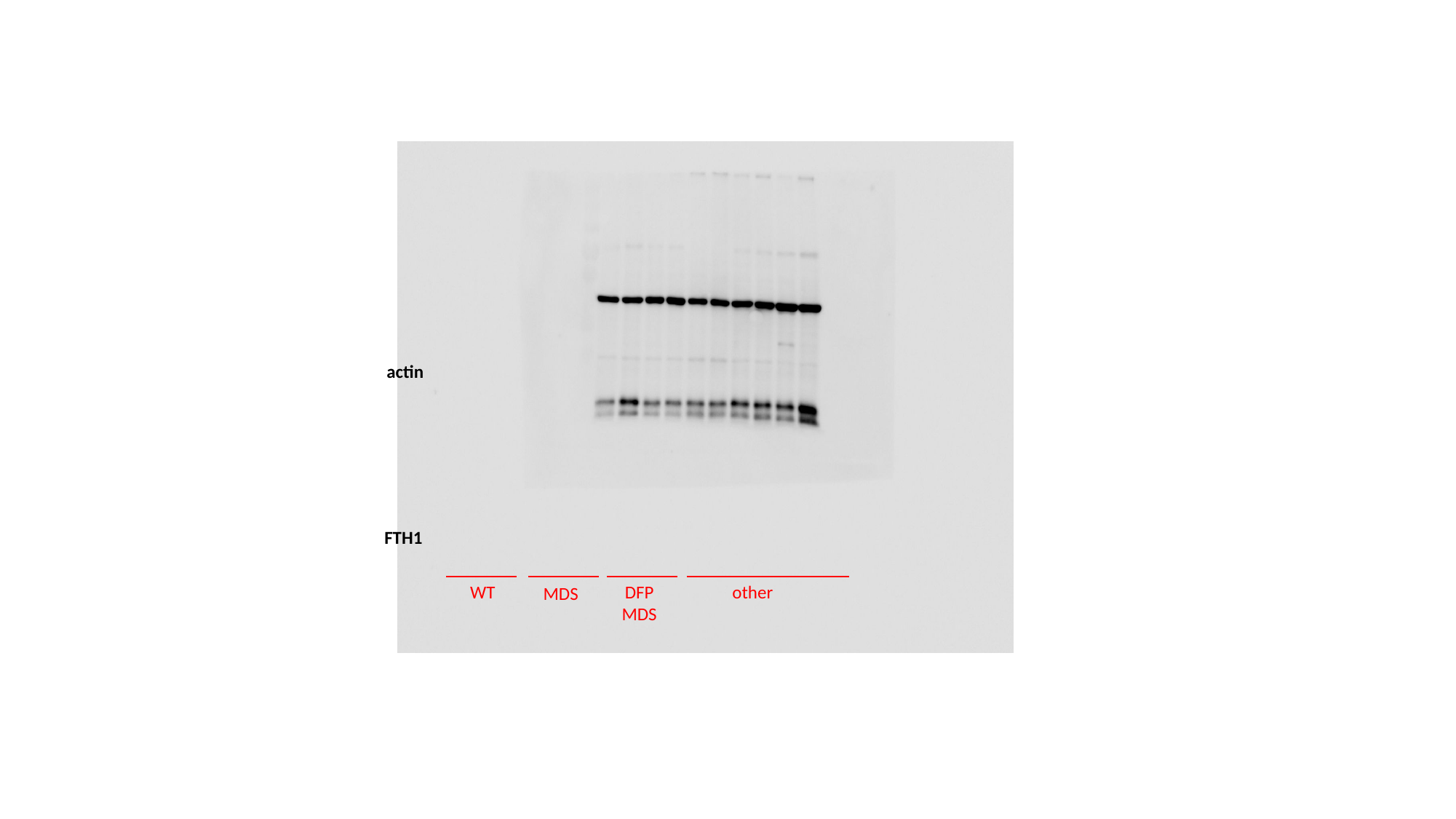

FTH1
WT
DFP MDS
other
MDS
actin

Supplement: Figure 1—figure supplement 4—source data 1. [file elife-83103-fig1-figsupp4-data1.zip › 83103R1 Figure 1 - figure supplement 4 source data 1 5.pptx]

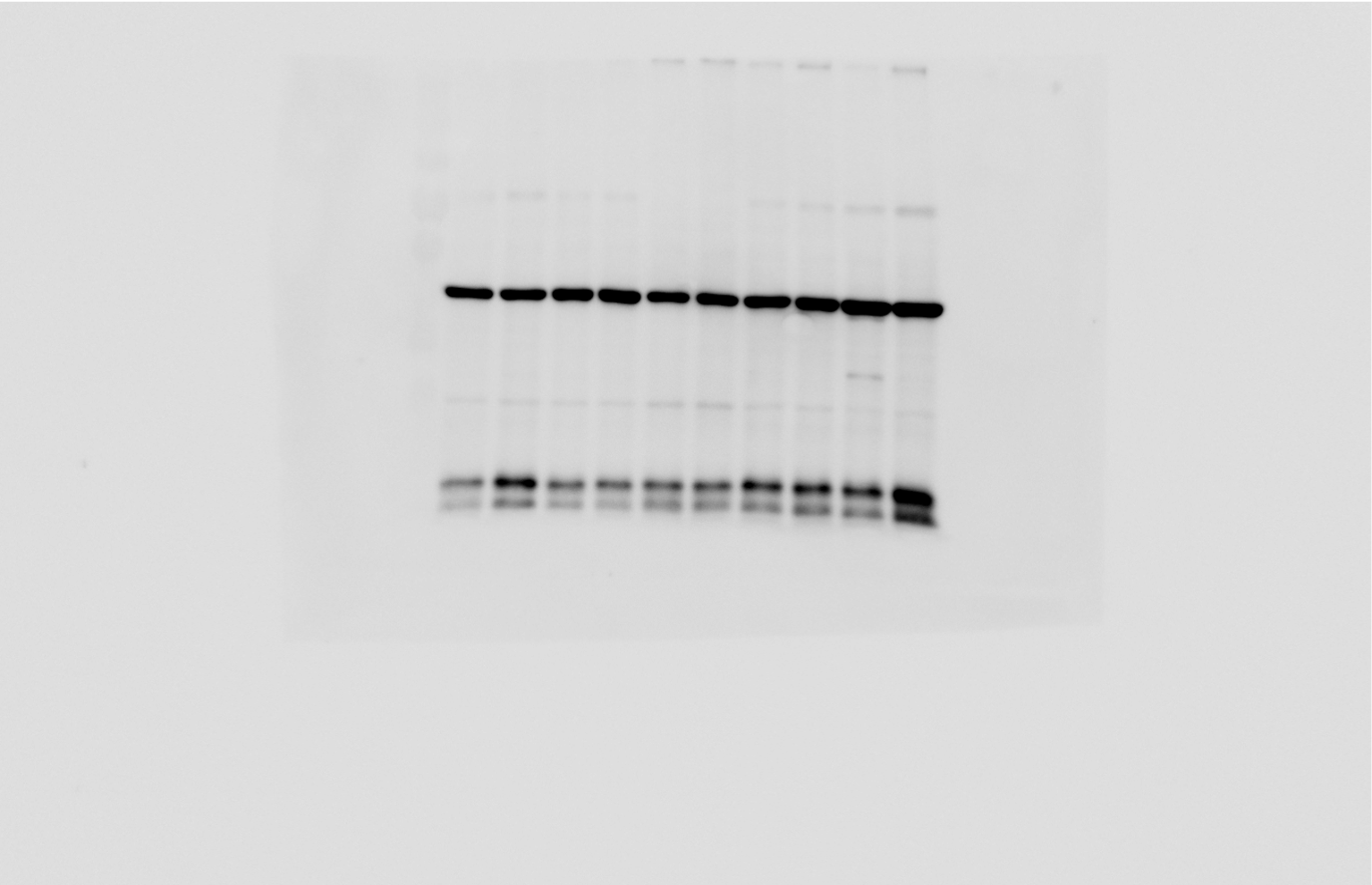

Supplement: Figure 1—figure supplement 4—source data 1. [file elife-83103-fig1-figsupp4-data1.zip › 83103R1 Figure 1 supplement 4 source data 1 (2).tif]

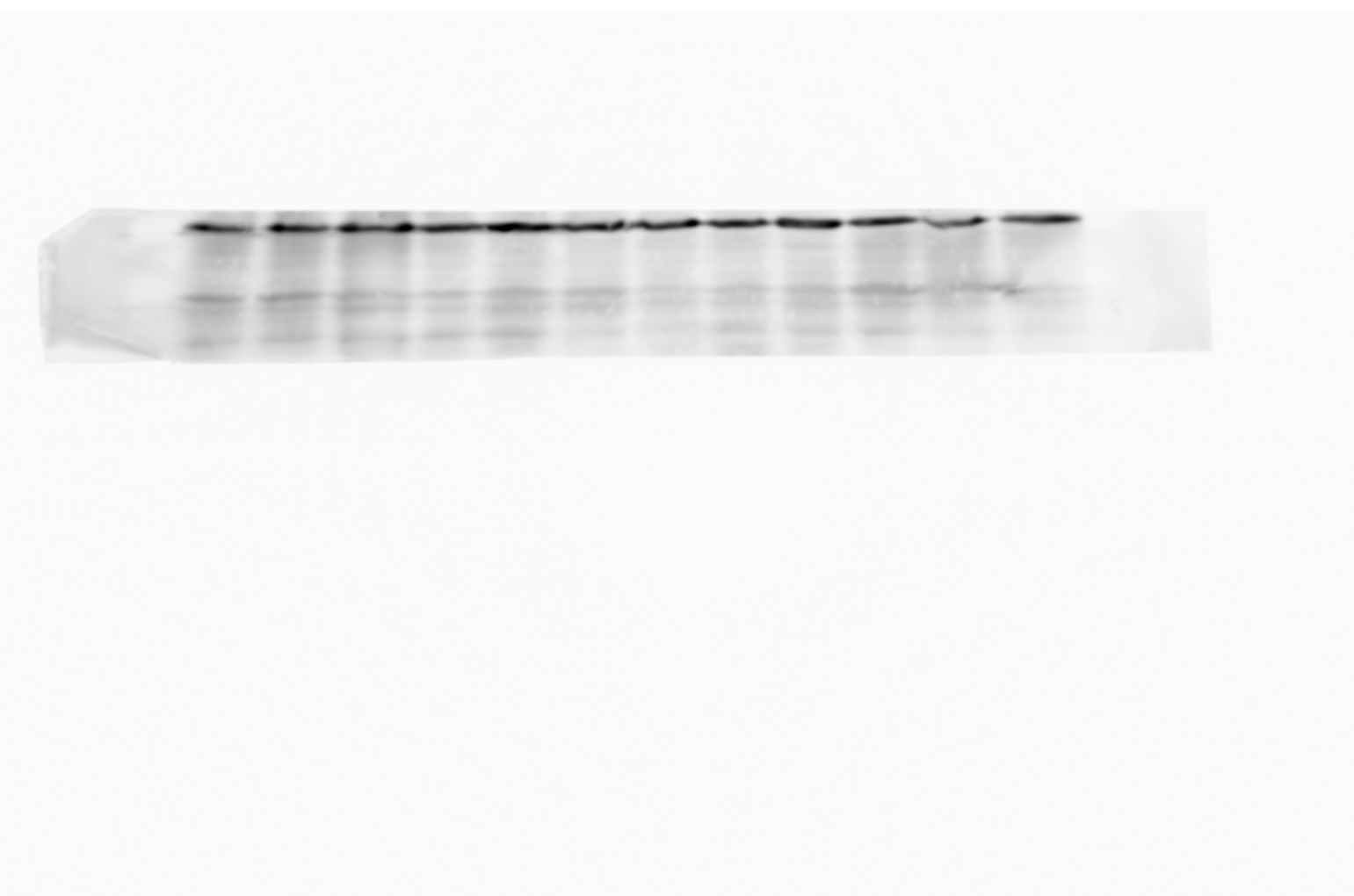

Supplement: Figure 1—figure supplement 5—source data 1. [file elife-83103-fig1-figsupp5-data1.zip › 83130r1 Figure 1 supplement 5 source data 1c (2).tif]

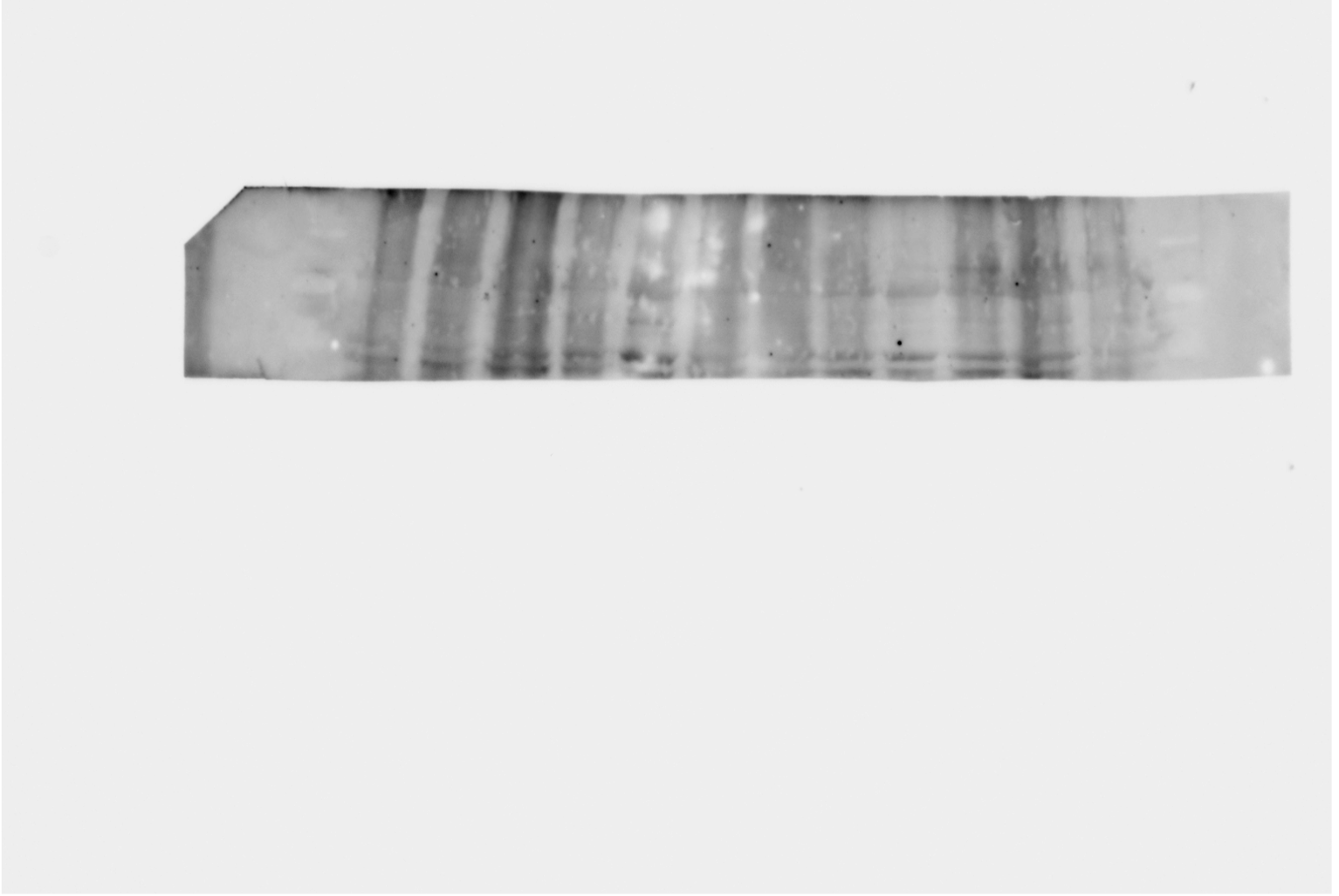

Supplement: Figure 1—figure supplement 5—source data 1. [file elife-83103-fig1-figsupp5-data1.zip › 83103r1 Figure 1 supplement 5 source data 1a (2).tif]

## Slide 1
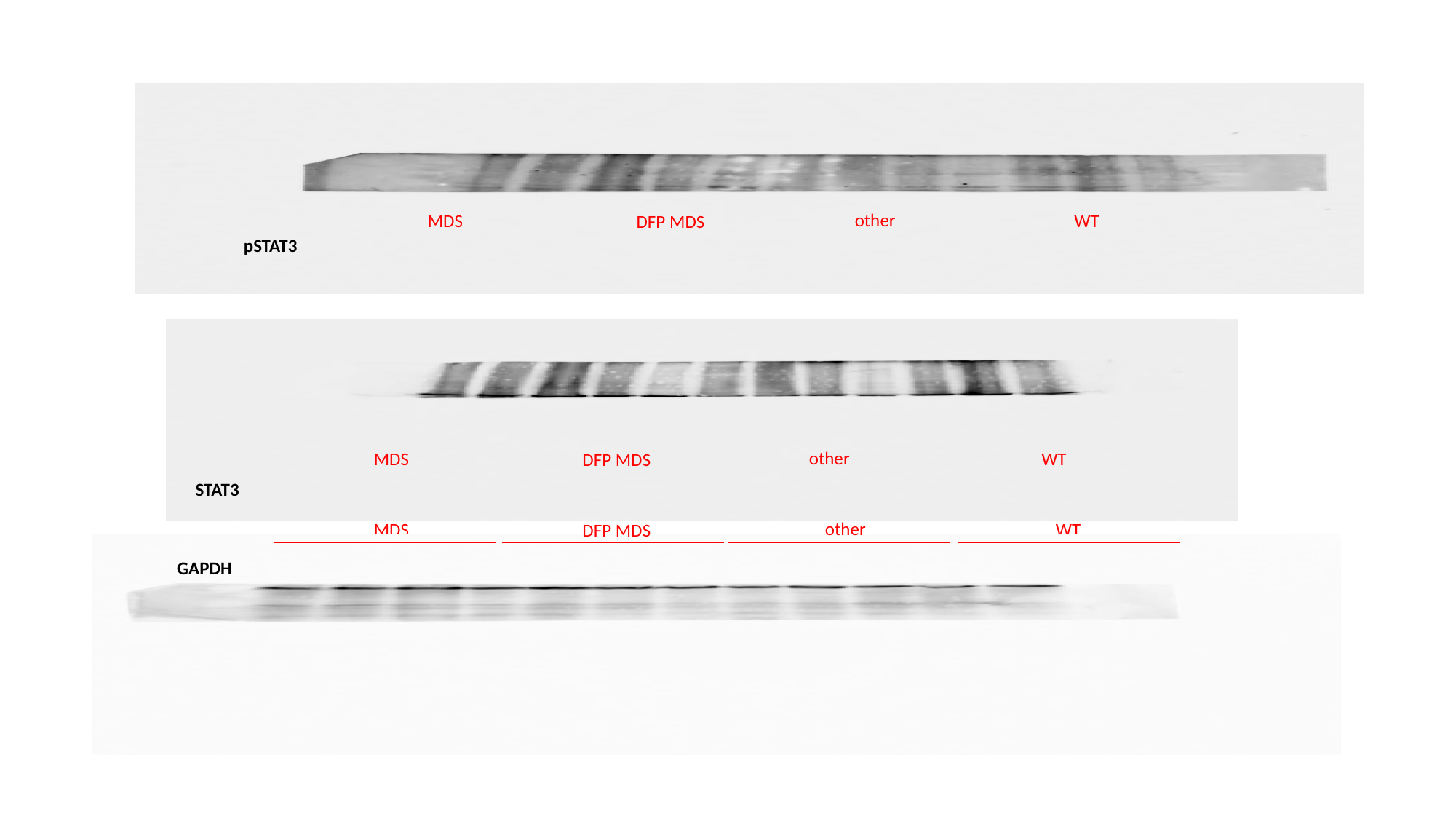

other
MDS
WT
DFP MDS
pSTAT3
other
MDS
WT
DFP MDS
STAT3
other
MDS
WT
DFP MDS
GAPDH

Supplement: Figure 1—figure supplement 5—source data 1. [file elife-83103-fig1-figsupp5-data1.zip › 83130R1 Figure 1 - figure supplement 5 source data 1.pptx]

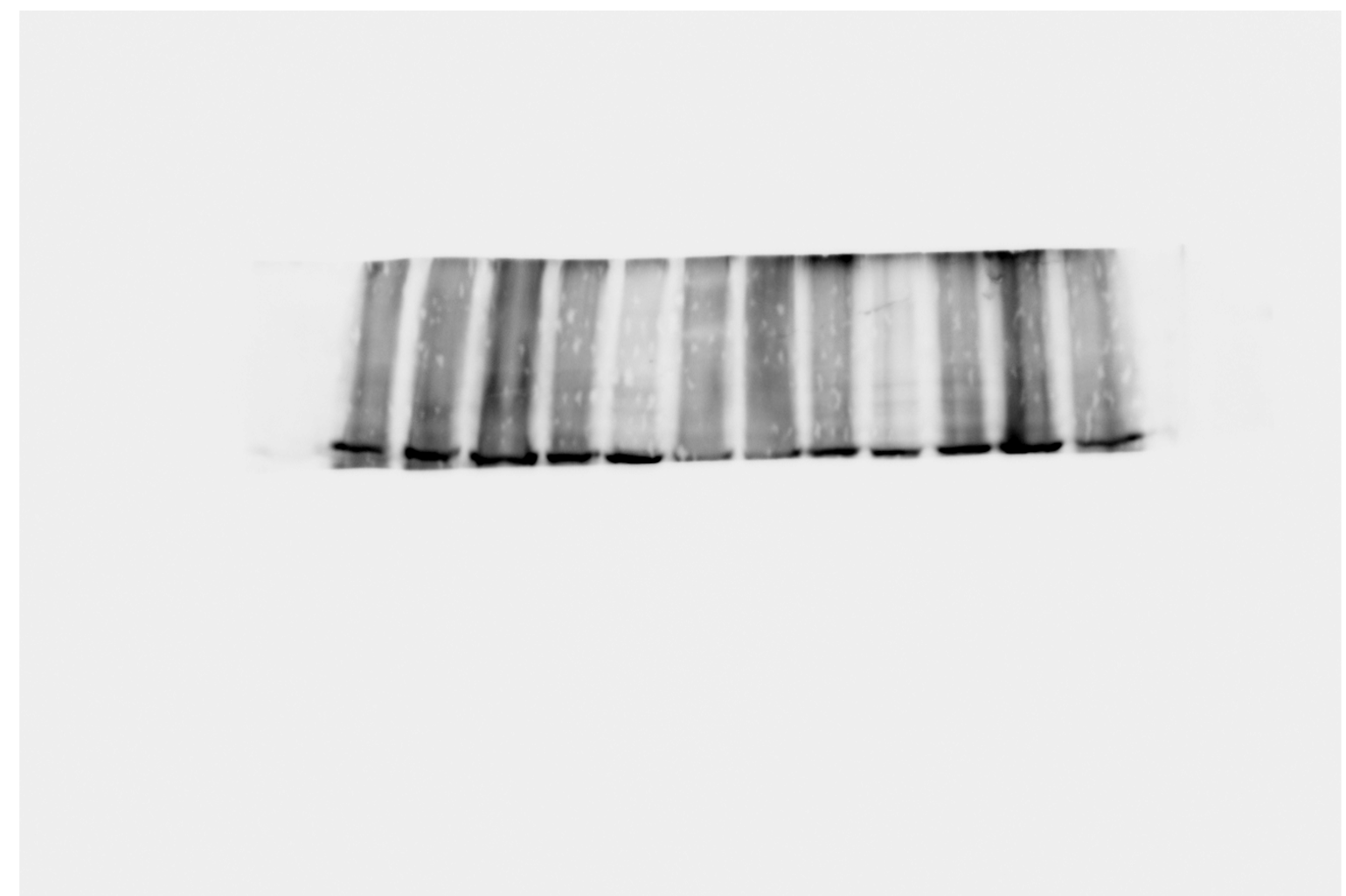

Supplement: Figure 1—figure supplement 5—source data 1. [file elife-83103-fig1-figsupp5-data1.zip › 83130r1 Figure 1 supplement 5 source data 1b (1).tif]

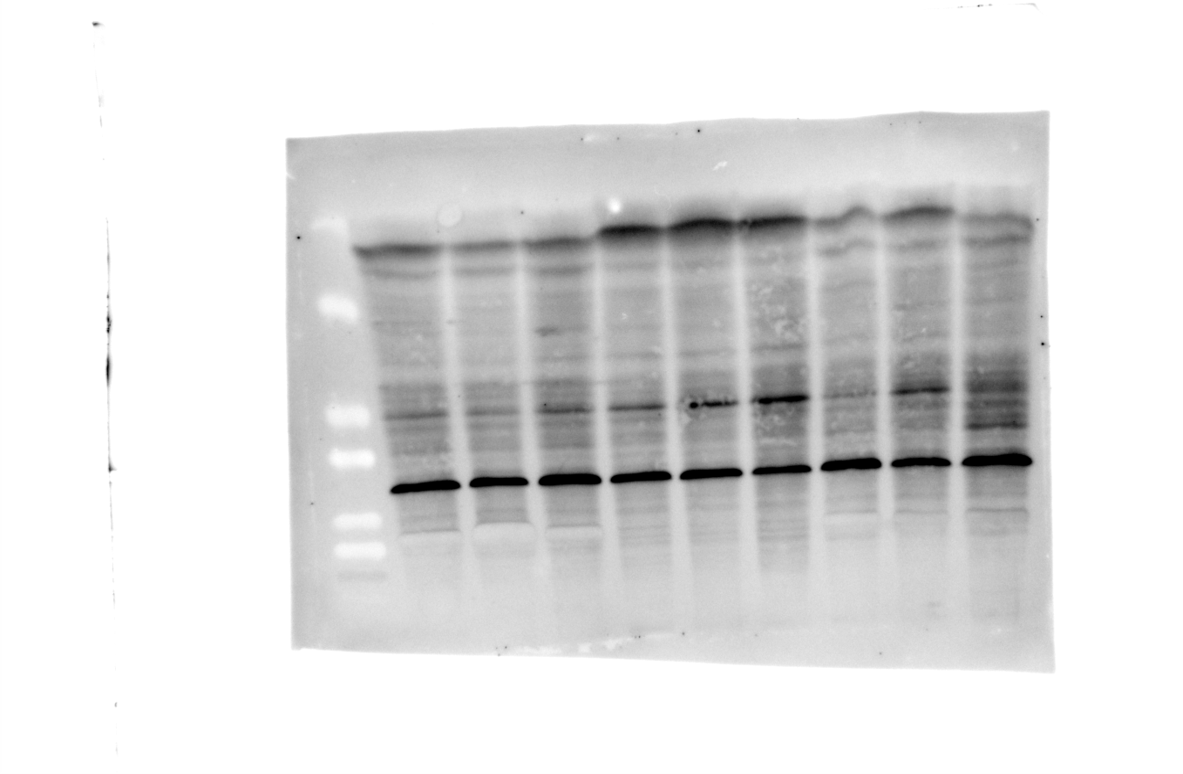

Supplement: Figure 4—figure supplement 2—source data 1. [file elife-83103-fig4-figsupp2-data1.zip › 83130 Figure 4 supplement 2 source data 1a (2).tif]

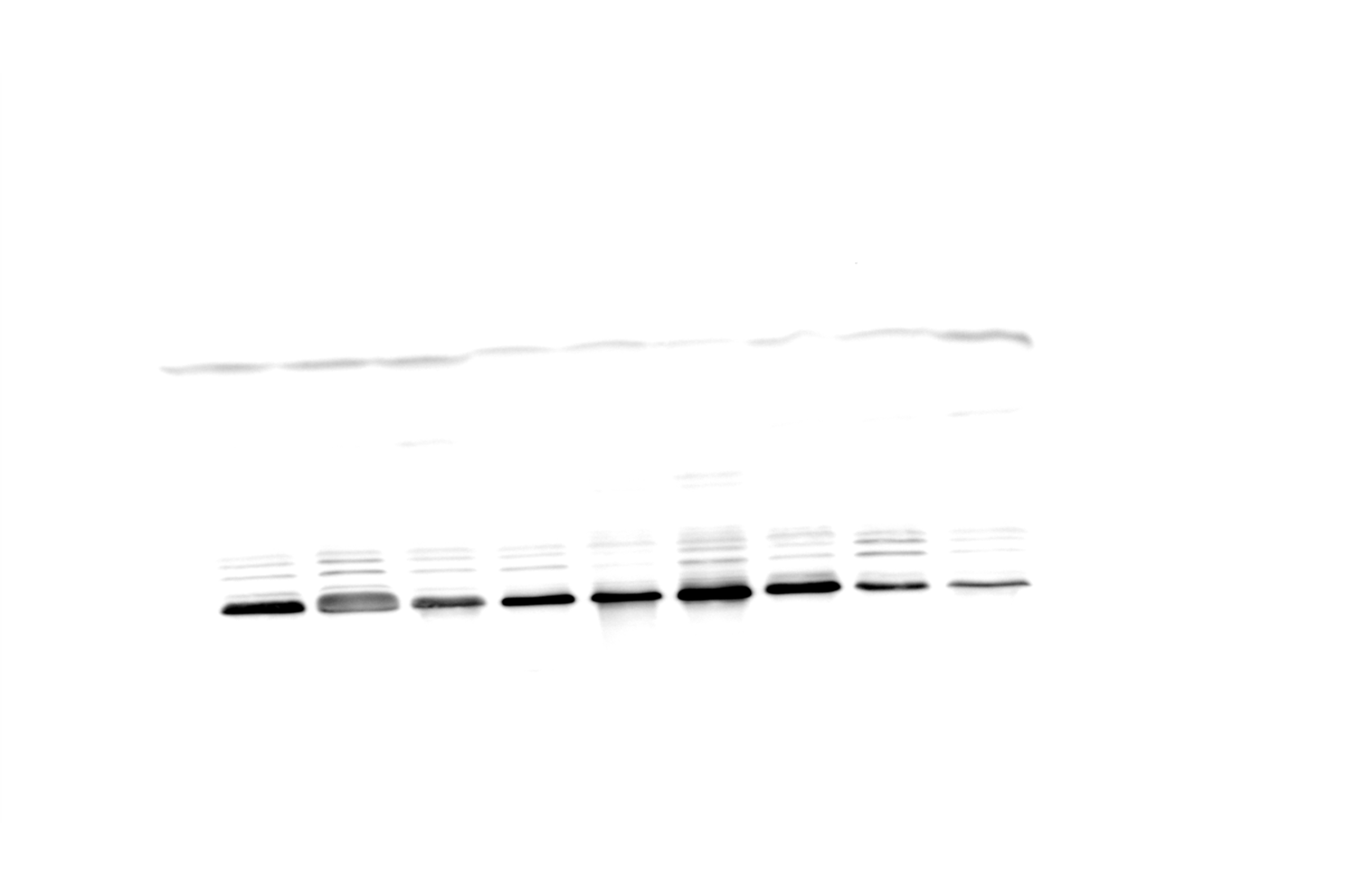

Supplement: Figure 4—figure supplement 2—source data 1. [file elife-83103-fig4-figsupp2-data1.zip › 83130 Figure 4 supplement 2 source data 1b (2).tif]

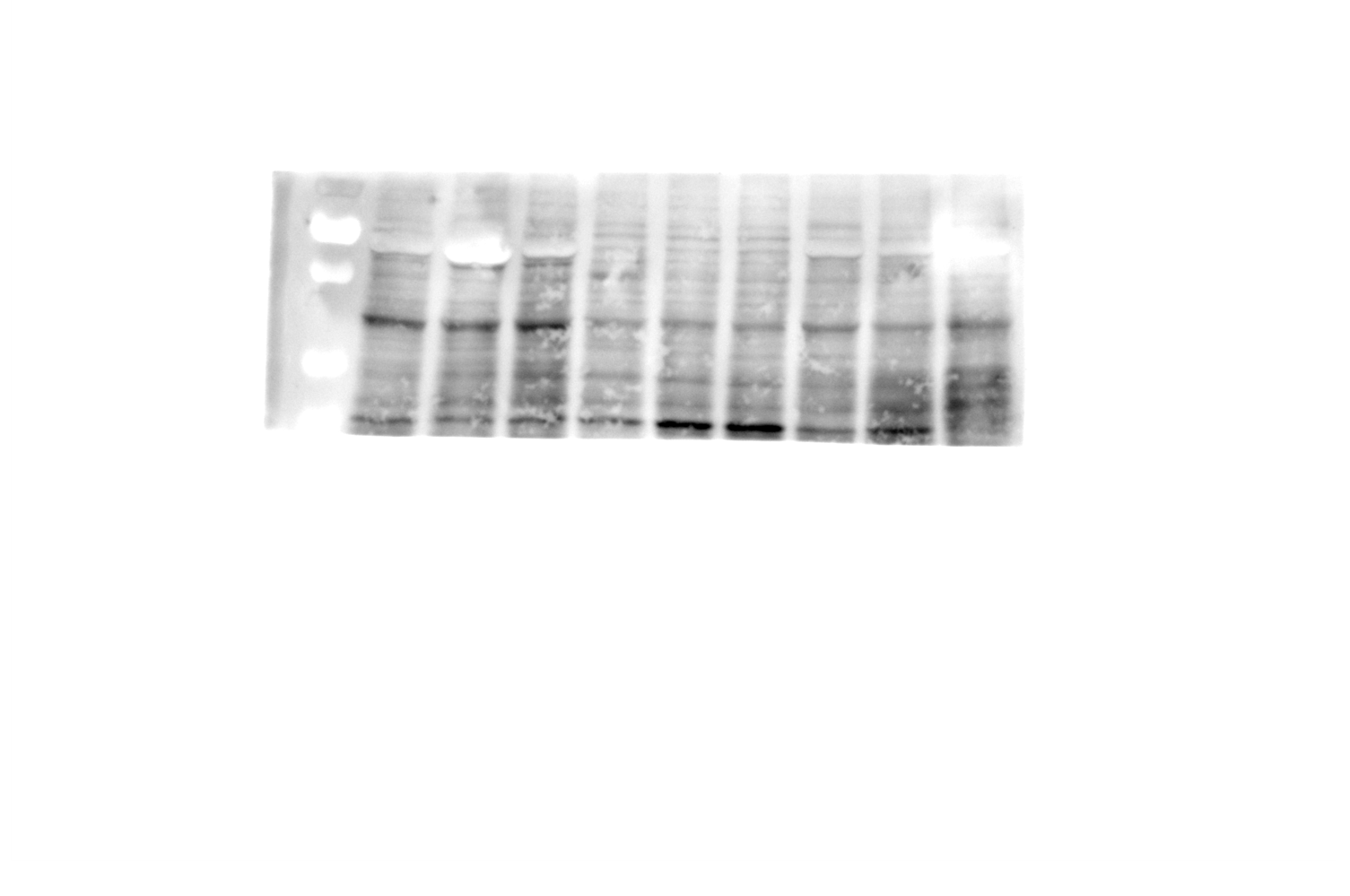

Supplement: Figure 4—figure supplement 2—source data 1. [file elife-83103-fig4-figsupp2-data1.zip › 83130 Figure 4 supplement 2 source data 1c (2).tif]

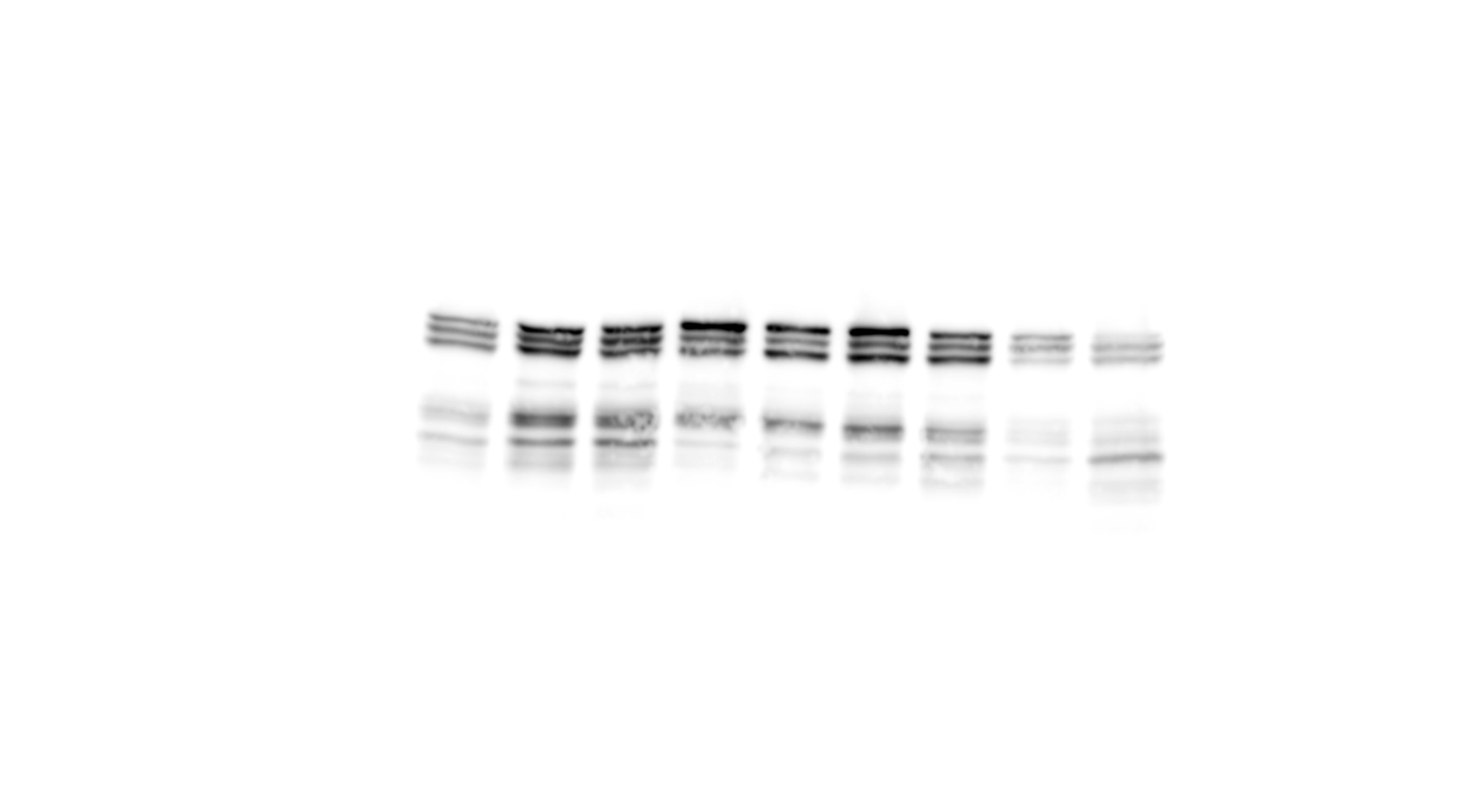

Supplement: Figure 4—figure supplement 2—source data 1. [file elife-83103-fig4-figsupp2-data1.zip › 83130 Figure 4 supplement 2 source data 1d (3).tif]

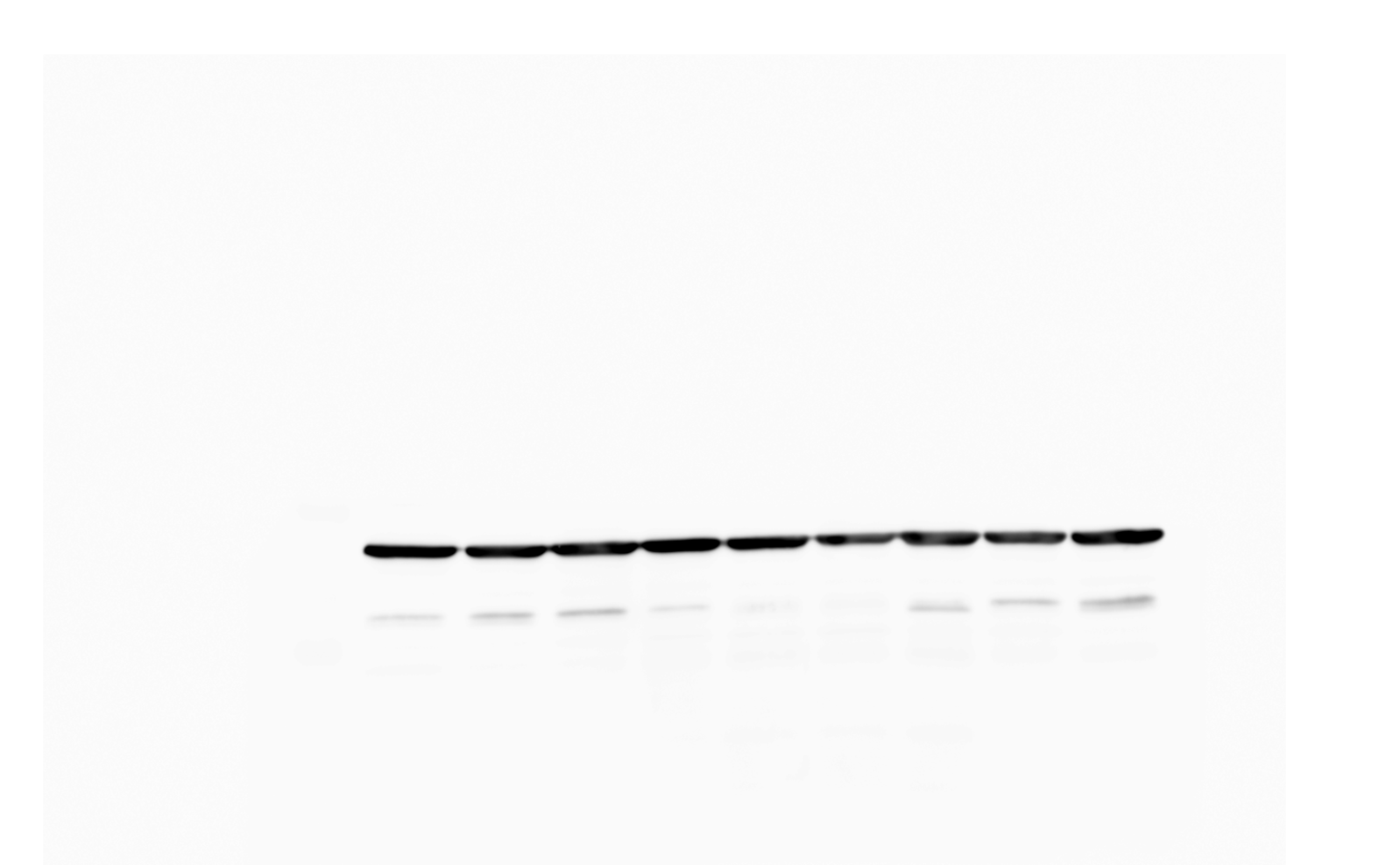

Supplement: Figure 4—figure supplement 2—source data 1. [file elife-83103-fig4-figsupp2-data1.zip › 83130 Figure 4 supplement 2 source data 1e (6).tif]

## Slide 1
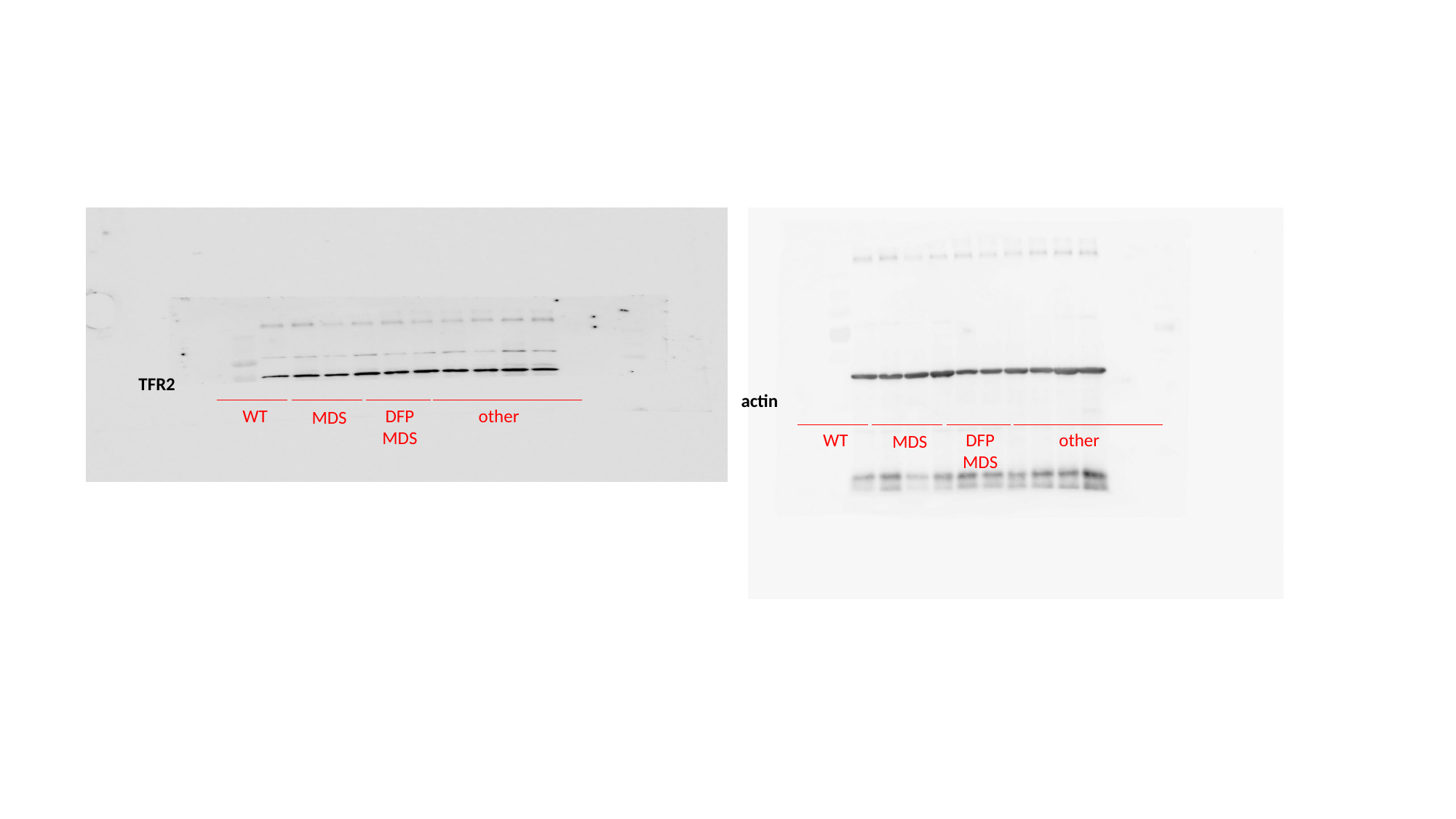

TFR2
actin
WT
DFP MDS
other
MDS
WT
DFP MDS
other
MDS

Supplement: Figure 7—source data 2. [file elife-83103-fig7-data2.zip › 83130 Figure 7 source data 2.pptx]

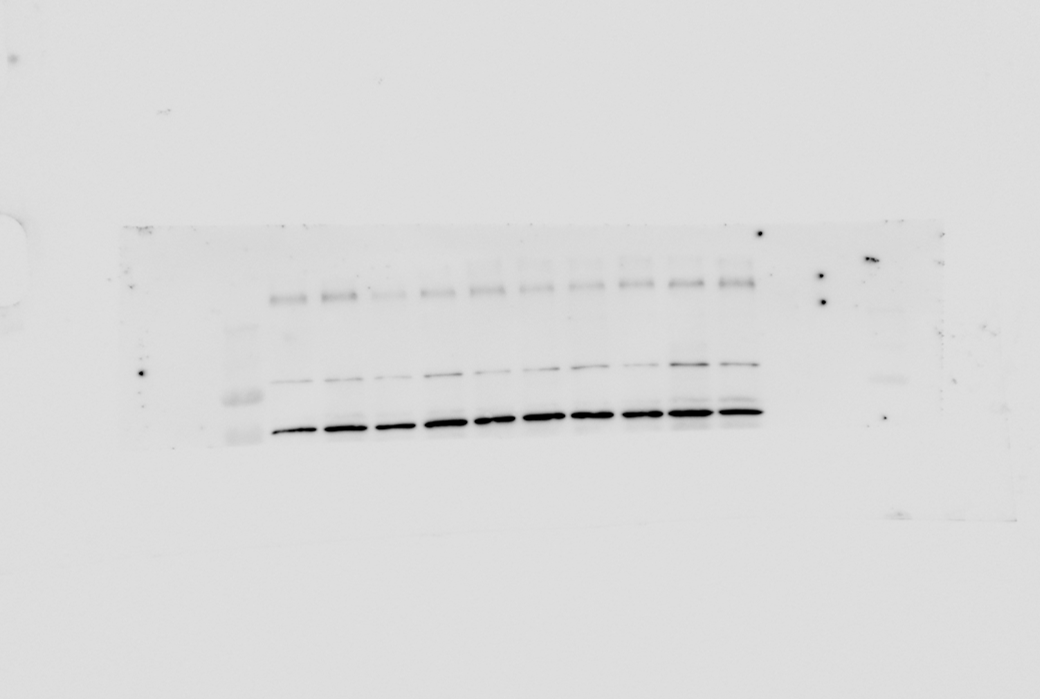

Supplement: Figure 7—source data 2. [file elife-83103-fig7-data2.zip › 83130 Figure 7 source data 2a (2).tif]

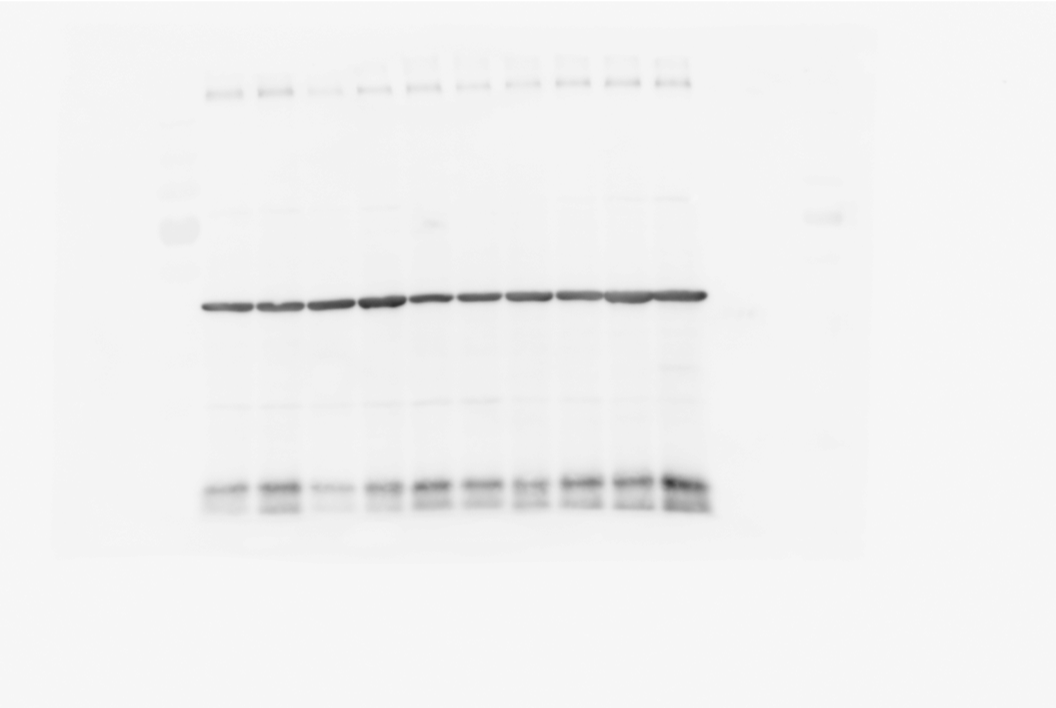

Supplement: Figure 7—source data 2. [file elife-83103-fig7-data2.zip › 83130 Figure 7 source data 2b (4).tif]
