# Supplementary material for: Iron chelation improves ineffective erythropoiesis and iron overload in myelodysplastic syndrome mice
Source: eLife. 2023 Dec 28;12:e83103. doi: 10.7554/eLife.83103 (PMC10754500; doi:10.7554/eLife.83103)
Supplement: Figure 4—figure supplement 2—source data 1. [file elife-83103-fig4-figsupp2-data1.zip › 83130 Figure 4 figure supplement 2 source data 1 - Copy.pptx]

## Slide 1
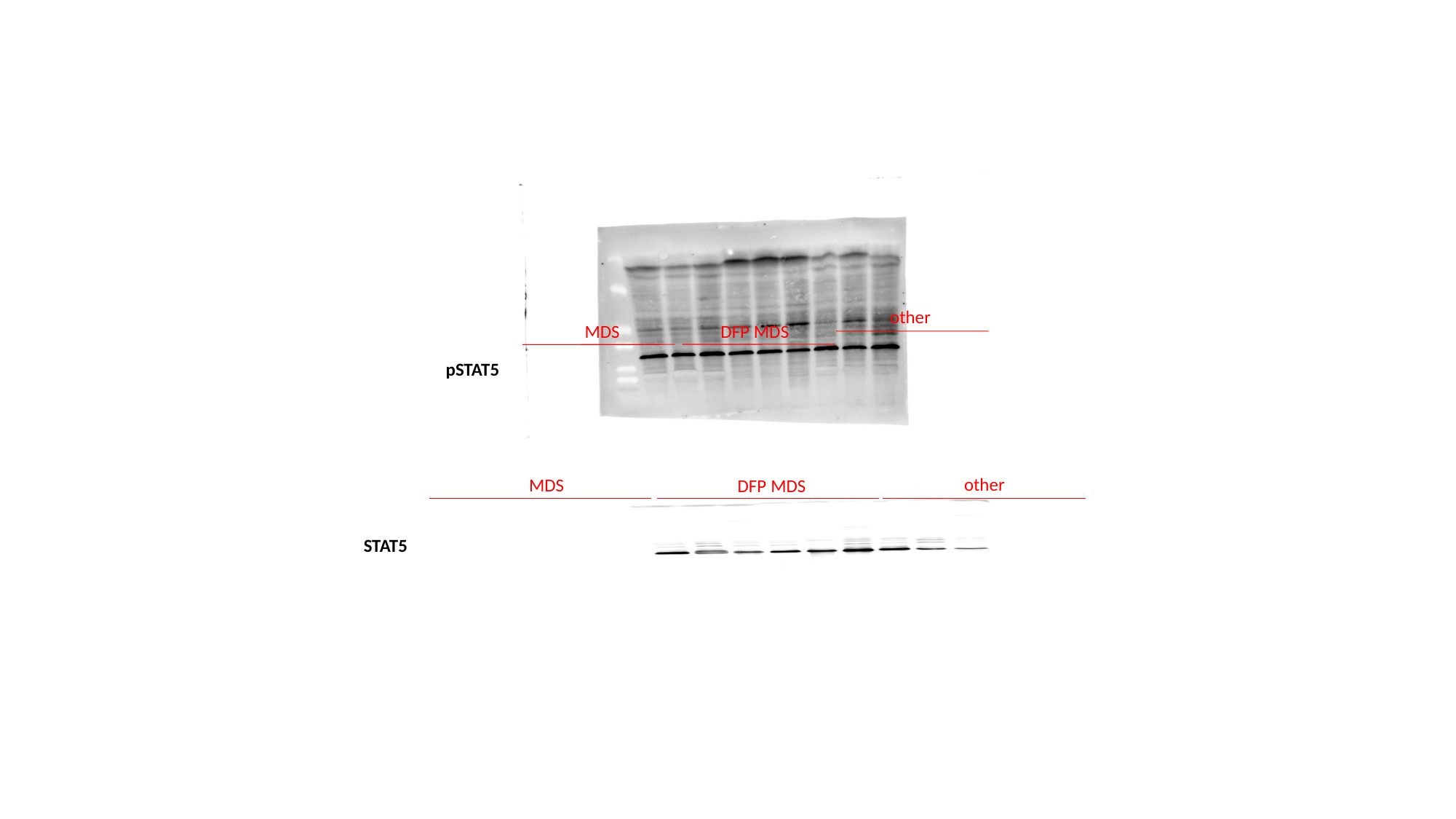

other
MDS
DFP MDS
pSTAT5
other
MDS
DFP MDS
STAT5

## Slide 2
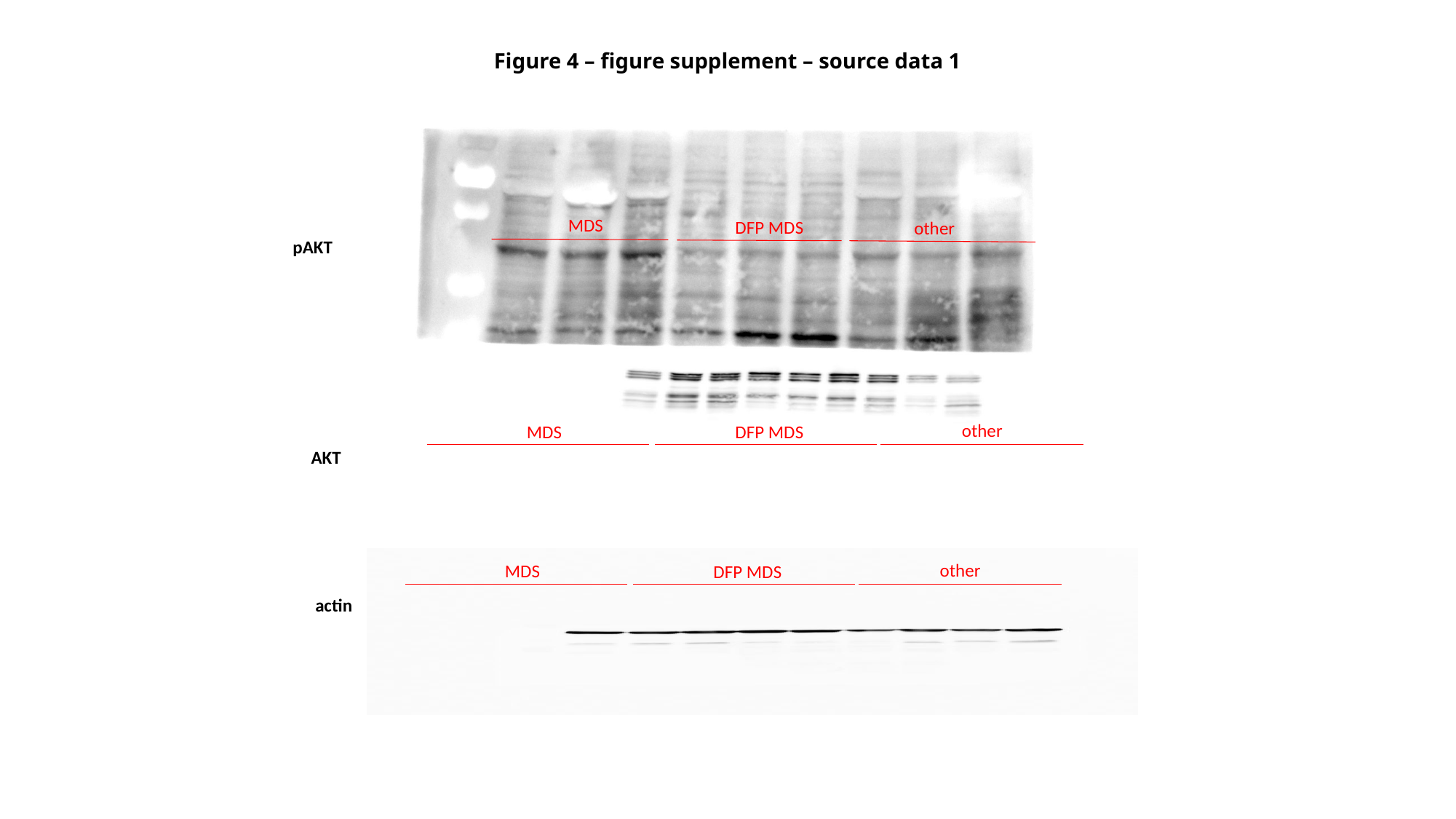

Figure 4 – figure supplement – source data 1
MDS
DFP MDS
other
pAKT
other
MDS
DFP MDS
AKT
other
MDS
DFP MDS
actin
