# Supplementary material for: Iron chelation improves ineffective erythropoiesis and iron overload in myelodysplastic syndrome mice
Source: eLife. 2023 Dec 28;12:e83103. doi: 10.7554/eLife.83103 (PMC10754500; doi:10.7554/eLife.83103)
Supplement: Supplementary file 1. — (a) Table of primers. (b) Table of antibodies. [file elife-83103-supp1.docx]

Supplementary File 1a

| **Gene** | **Forward (sense)** | **Reverse (antisense)** |
| --- | --- | --- |
| *mErfe* | ATGGGGCTGGAGAACAGC | TGGCATTGTCCAAGAAGACA |
| *mTfrc* | ATAAGCTTTGGGTGGGAGGC | TGGTTCCCCACCAAACAAGT |
| *mTfr2* | CCTGATCACCCTGCTAATCTTC | TCTTCATCGACCACCAACAC |
| *mPcbp1* | AATCAATGCCAGGCTTTCCTC | TTAAAACCTGGAATTACCGACCAG |
| *mPcbp2* | AATCAATGCCAGGCTTTCCTC | TTAAAACCTGGAATCGCTGACTG |
| *mNcoa4* | TGGTTGGTGACTCCTCAGGAA | TCACTCACATTGTAGGGCTCT |
| *mGpx4* | CCTCTGCTGCAAGAGCCTCCC | CTTATCCAGGCAGACCATGTGC |
| *mBcl11a* | GACAAGGAGATGCAGGTATTGG | TCCCGTAGAGATCCACAAAAGT |
| *mGata1* | CGTCATACCACTAAGGTGGCTGAAT | GTGGAATCTGATGGTGAGGACA |
| *mScrib* | GGGGTGATCCAGCCATTGG | GGCCCTATACGCCTGCTTC |
| *mEpor* | TCATACCAGCTCGAGGGTGA | GGTGATAGCGAGGAGAACCG |
| *mHamp* | CTGAGCAGCACCACCTATCTC | TGGCTCTAGGCTATGTTTTGC |
| *mSaa1* | AGTCTGGGGTGCTGAGAAAA | ATGTCTGTTGGCTTCCTGGT |
| *mGapdh* | AACAGCAACTCCCACTCTTC | CCTGTTGCTGTAGCCGTATT |

Supplementary File 1b

| **Antibody** | **Company** | **Catalog #** | **Reference** |
| --- | --- | --- | --- |
| Beta-actin | ThermoFisher | MA515452 |  |
| GAPDH | Cell Signaling Technology | 97166 |  |
| Phospho-STAT3 | Cell Signaling Technology | 9131 | Tsagianni *et al*., 2018 |
| STAT3 | Cell Signaling Technology | 12640 | Shi *et al*., 2016 |
| Phospho-STAT5 | Cell Signaling Technology | 9359 | Shi *et al*., 2016 |
| STAT5 | Cell Signaling Technology | 94205 | Niogret *et al*., 2019 |
| Phospho-AKT | Cell Signaling Technology | 4058 | Caffa *et al*., 2020 |
| AKT | Cell Signaling Technology | 4691 | Reddy *et al*., 2019 |
| FTH1 | Cell Signaling Technology | 4393 | Santana-Codina *et al*., 2019 |
| TFR2 | Abcam | ab80194 | Berezovsky *et al*., 2021 |
